# Can the validity of a cohort be improved by reweighting based on register data? Evidence from the Swedish MDC study

**DOI:** 10.1186/s12889-020-10004-z

**Published:** 2020-12-17

**Authors:** Anton Nilsson, Carl Bonander, Ulf Strömberg, Jonas Björk

**Affiliations:** 1 Epidemiology, Population studies and Infrastructures (EPI@LUND), Tornblad Building, Lund University, Biskopsgatan 9, Hämtställe 21, SE-22362 Lund, Sweden; 2 Centre for Economic Demography, Lund University, Lund, Sweden; 3 Health Economics and Policy, University of Gothenburg, Gothenburg, Sweden; 4 Region Halland, Halmstad, Sweden; 5 Clinical Studies Sweden, Forum South, Skåne University Hospital, Lund, Sweden

**Keywords:** Validity, Representativeness, Reweighting, Mortality, Incidence, Cohorts

## Abstract

**Background:**

In any study with voluntary participation, self-selection risks leading to invalid conclusions. If the determinants of selection are observed, it is however possible to restore the parameters of interest by reweighting the sample to match the population, but this approach has seldom been applied in epidemiological research.

**Methods:**

We reweighted the Malmö Diet and Cancer (MDC) study based on population register data on background variables, including socio-demographics and hospital admissions for both participants and the background population. Following individuals from baseline in 1991–1996 and at most until 2016, we studied mortality (all-cause, cancer, and CVD), incidences (cancer and CVD), and associations between these outcomes and background variables. Results from the unweighted and reweighted participant sample were compared with those from the background population.

**Results:**

Mortality was substantially lower in participants than in the background population, but reweighting the sample helped only little to make the numbers similar to those in the background population. For incidences and associations, numbers were generally similar between participants and the background population already without reweighting, rendering reweighting unnecessary.

**Conclusion:**

Reweighting samples based on an extensive range of sociodemographic characteristics and previous hospitalizations does not necessarily yield results that are valid for the population as a whole. In the case of MDC, there appear to be important factors related to both mortality and selection into the study that are not observable in registry data, making it difficult to obtain accurate numbers on population mortality based on cohort participants. These issues seem less relevant for incidences and associations, however. Overall, our results suggest that representativeness must be judged on a case-by-case basis.

**Supplementary Information:**

The online version contains supplementary material available at 10.1186/s12889-020-10004-z.

## Background

Selective participation is a concern in most cohort studies. In particular, conclusions about prevalences, incidences, and associations may not generalize from the study sample to the population as a whole when participants are not representative of the full population [1–9]. Furthermore, the interpretation of associations as causal effects may be hampered [6, 10, 11]. While non-response and refusal to enroll in scientific studies have become increasing problems in both social science and medicine, few epidemiological studies have so far taken measures to correct for the implied biases.

In many ways, selection into a cohort resembles selection into an exposure, i.e., the phenomenon that can give rise to confounding. There is a recent theoretical literature on how representativeness can be restored by reweighting the cohort, particularly by using the method of inverse probability of participation weighting (IPPW) [12–15], a method with similarities to propensity score (PS) weighting to deal with confounding. To apply IPPW or similar methods, however, data on background variables for both participants and non-participants are required. Since such data have typically not been available for non-participants, there have so far been limited opportunities to evaluate the practical consequences of reweighting for representativeness.

Summarizing previous evidence on cohort participation in epidemiology, Galea and Tracy [16] noted that women, married, and individuals of higher socioeconomic status were more likely to participate. Other personal characteristics such as age or ethnicity have sometimes also been linked to participation, but with no consistent pattern across studies. In any case, given the strong relationship between socioeconomic status and health [17–19], one may often expect individuals with poor health to be underrepresented in studies. In a previous article based on another Swedish cohort study and where it was possible to compare prevalences in the sample with those in the population, we showed that reweighting based on sociodemographic register data worked very well and sometimes appeared sufficient to correct for selection into the cohort [7].

In this article, we considered selection into the Malmö Diet and Cancer (MDC) study, a cohort study conducted in Southern Sweden with a baseline examination including a self-administered questionnaire, anthropometric measuring, and blood sampling [20]. Recruitment to this cohort took place between 1991 and 1996, with a participation rate of about 40%. As has been shown previously, participants and non-participants differed in terms of cancer incidence and mortality, both before and during recruitment, and during a short follow-up period [21]. In the present study, we investigated the possibilities to use reweighting to correct for selective participation in a long-term follow-up, considering outcomes such as mortality, disease incidences, and associations with these outcomes, using weights based on socio-demographics and disease history. As we had access to data on the same outcomes also in the background population, we were able to evaluate to what extent the reweighting method was able to improve representativeness of the study cohort.

## Methods

### Data analysis

We reweighted the sample with a method in line with previous literature on IPPW weighting [7, 12–14].A special feature of our data, however, was that it came in two separate sets – one for the participants and one for the full background population, with no linkage or indicator of who in the background population was a participant. As a result, the standard IPPW method could not be applied. Instead, we used an approach similar to that applied in studies of transportability, where the aim is normally to produce estimates that are valid for an entirely different population than the background population [12, 22]. As we have described in a previous article, this approach can also be used to achieve generalizability in situations like ours, where data on participants and the background population come in separate datasets [23].

As a first step, we combined the participant sample and the background population datasets into one dataset, where each participant thus appeared twice (but could only be identified as a participant once). A binary variable was created to indicate membership in the participant sample, and a logistic regression was then applied to predict this membership based on the background characteristics, including socio-demographics and disease history. As we have shown, predicted odds for belonging to the participant sample could then be interpreted as predicted probabilities of actually being a participant [23]. Sampling weights were calculated for the participants as the multiplicative inverses of the predicted probabilities of actually being a participant.

The distributions of background characteristics in the background population were compared with those in the participant sample and with those in the reweighted participant sample. (In principle, background characteristics in the reweighted participant sample should resemble those in the background population, as the reweighting was made exactly based on these.) Subsequent mortality and incidence of hospitalization were then compared across the background population, participants, and reweighted participants. Additional analyses were stratified on quintiles of the estimated participation probabilities, allowing us to examine if differences between participants and individuals in the background population may have been concentrated, for example, to those with a low propensity to participate. Furthermore, we used Cox regressions to estimate associations between outcomes and background variables, and to compare these across the background population, participants, and reweighted participants.

To evaluate the ability of the logistic regression model to predict participation based on the background variables, the area under the ROC curve was calculated. We also visually examined the estimated participation probabilities, separately for participants and non-participants. In these two analyses, duplicates of participants were removed by sorting the data according to the predicted probabilities of participating, and for each predicted probability omitting the same number of individuals from the background population as the number of individuals in the participant sample. Analyses were performed using Stata 15.1 (StataCorp) and SPSS Statistics 25 (IBM).

### Data

Our full background population consisted of all men (born 1923–1945) and women (born 1923–1950) who lived in Malmö between January 1, 1991, and September, 30, 1996. This population, comprising 74,103 individuals, essentially corresponded to those who were invited to participate in the MDC study. (In practice, some were never invited because of death, migration, or other issues.) The participant sample comprised 28,098 individuals. All participants in MDC provided written informed consent at enrollment.

Data on socio-demographics were retrieved from Statistics Sweden and spanned the years 1990 to 2016. These data included year of birth, sex, civil status, country of birth (grouped), migration events, and an array of socioeconomic information, such as the highest level of education and income from different sources. Moreover, we retrieved data from the National Board of Health and Welfare, including the Patient Register, covering all hospitalizations and associated diagnoses from 1987 to 2016, and the Cause of Death Register, from which we obtained data on deaths and causes of deaths between 1990 and 2016. The reason for retrieving hospitalizations specifically from 1987 was that the Patient Register reached national coverage in this year, and we wished to account for hospitalizations during at least a few years prior to baseline.

For participants in MDC, we made use of background data on socio-demographics in the year prior to enrollment (or in the same year if no data was available in the previous year, which could occur if the individual had lived abroad). Hospitalizations were divided into groups based on the International Classification of Diseases (ICD), version 9 or 10: neoplasms (ICD codes 140–239/C00-D48), diabetes (ICD codes 250/E10-E14), mental and behavioral disorders (ICD codes 290–319/F00-F99), diseases of the circulatory system (ICD codes 390–459/I00-I99), diseases of the respiratory system (ICD codes 460–519/J00-J99), and diseases of the digestive system (ICD codes 520–579/K00-K93). Binary indicators were created to measure if the individual had had at least one hospitalization for these types of diagnoses between 1987 and enrollment.

There was no information on when individuals in the background population were invited to participate in the MDC study. We therefore assigned “imaginary” dates of enrollment to individuals in the background population, where the calendar date was always set to July 1 and the enrollment year was drawn from the birth-year-specific distribution of enrollment years observed among participants. Individuals in the background population who had moved, died, or for other reasons lacked information on sociodemographic variables around the time of imaginary enrollment were excluded, reducing the background population to 71,447 individuals. Among the 28,098 participants, two were excluded because there was no information on sociodemographic variables around the time of enrollment.

Outcomes examined included mortality and incidence of disease. We considered all-cause mortality, but also the two most common causes of death: deaths due to cardiovascular disease (CVD) and cancer. Furthermore, we considered incidences of CVD and cancer. Following previous studies on MDC [24], CVD mortality was defined by ICD codes 390–459/I00–99 whereas incident CVD was defined as the occurrence of either a coronary event (a fatal or nonfatal myocardial infarction, 410/I21, or a death due to ischemic heart disease, 414/414/I22/I23/I25) or a fatal or nonfatal stroke (430/431/434/436/I60/I61/I63/I64), whichever came first. Incident cancer was conventionally defined by ICD codes 140–209/C00–99.

Individuals who had not yet experienced the particular outcome of interest were followed from the time of baseline examination (or imaginary enrollment) in MDC, and contributed with person-time until the first event of interest occurred, or until death or emigration; at most until the end of 2016.

## Results

Table 1 displays background characteristics, reported separately for the full background population and for the participants. It also includes the same descriptive statistics for the reweighed participant sample. Compared to the background population, individuals in the unweighted participant sample were more likely to be aged 56–64, female, born in Sweden, married, have more than primary education, be employed, and have higher income. The differences were mainly noticeable for country of birth and the different aspects of socioeconomic status. For disease history, the difference was mainly that participants were less likely to have had a hospitalization for a mental or behavioral disorder.

**Table 1:** Background characteristics (%), in the background population and in the participant sample, before and after reweighting

	Population (*n* = 71,447)	Participants (*n* = 28,096)	Participants, reweighted
*Socio-demographics*			
Age			
40–45	11	10	11
46–50	23	21	23
51–55	19	19	19
56–60	18	18	18
61–64	19	22	19
65–67	10	9.8	10
Female	58	61	58
Country of birth			
Sweden	79	88	80
Other Nordic	4.7	4.0	4.8
Other EU15	3.0	2.5	3.0
Other EU	5.3	2.9	5.2
Other Europe	4.7	1.5	4.5
Outside Europe	3.0	1.1	2.7
Civil status			
Married	60	66	60
Unmarried	12	9.3	12
Divorced	21	18	21
Widowed	7.3	6.9	6.9
Education			
Primary	44	37	42
Short secondary	27	29	28
Long secondary	11	13	11
Tertiary	18	21	19
Employment status			
Employed	55	63	56
Unemployed	9.0	5.6	8.1
Sickness absence	5.2	4.5	5.3
Retired	31	27	30
Disposable income			
Quintile 1	20	15	19
Quintile 2	20	17	20
Quintile 3	20	21	20
Quintile 4	20	23	21
Quintile 5	20	24	21
*Disease history*			
Circulatory	8.2	7.3	8.2
Diabetes	1.2	0.80	1.2
Neoplasms	5.2	5.4	5.4
Respiratory	2.9	2.4	2.9
Digestive	7.2	6.9	7.1
Mental	4.1	2.1	4.1

Age refers to age in 1990. Disposable income was adjusted for inflation using the KPI index from Statistics Sweden. Disease history assumes the value of one if the individual was hospitalized for the disease type in question between 1987 and the baseline examinations in the MDC study

**Fig. 1:**
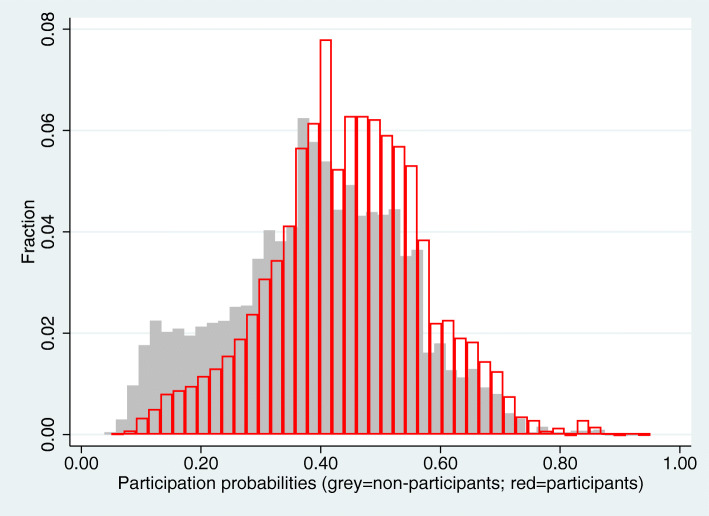
Histogram of estimated participation probabilities (obtained as estimated odds from a logistic regression), separated by participants and non-participants

Reweighting the participant sample produced distributions of background characteristics that throughout resembled those in the background population. The joint ability of the background characteristics to classify individuals as participants was, however, relatively modest (area under the ROC curve = 66.6%). Disease history contributed very little to the classifying ability (omitting previous hospitalizations only reduced the area under the ROC curve to 66.2%). Figure 1 shows the distribution of estimated participation probabilities, separately for participants and non-participants. While the distributions of the estimated probabilities overlapped, the central tendency was clearly higher among participants.

In Table 2, we display mortality and disease incidences in the background population, the participant sample, and the reweighted participant sample. Mortality was clearly higher in the background population than among participants, but less so for cancer mortality than for all-cause and CVD mortality. Reweighting only eliminated smaller shares of the gaps: 14% for all-cause mortality and 21% for CVD mortality. For cancer mortality, reweighting made virtually no difference.

**Table 2:** Mortality and disease incidences (events per 10,000 person-years) in the background population and in the participant sample, before and after reweighting; the follow-up begins at baseline in 1991–1996 and ends at an event, death, emigration, or at the latest in the end of 2016

	Population (n = 71,447)	Participants (n = 28,096)	Participants, reweighted
All-cause mortality			
Everyone	222	176	182
Participation quintile 1	264	196	192
Participation quintile 2	283	227	226
Participation quintile 3	223	188	188
Participation quintile 4	182	159	159
Participation quintile 5	171	150	150
CVD mortality			
Everyone	76	58	62
Participation quintile 1	94	72	71
Participation quintile 2	103	82	82
Participation quintile 3	76	64	64
Participation quintile 4	60	48	48
Participation quintile 5	53	46	46
Cancer mortality			
Everyone	72	63	64
Participation quintile 1	70	63	61
Participation quintile 2	84	71	70
Participation quintile 3	74	66	66
Participation quintile 4	67	62	62
Participation quintile 5	66	58	58
CVD incidence			
Everyone	128	113	118
Participation quintile 1	145	129	129
Participation quintile 2	154	144	144
Participation quintile 3	130	122	122
Participation quintile 4	111	99	100
Participation quintile 5	105	99	100
Cancer incidence			
Everyone	136	133	132
Participation quintile 1	119	120	117
Participation quintile 2	149	140	140
Participation quintile 3	137	133	133
Participation quintile 4	133	133	133
Participation quintile 5	140	134	134

Participation quintiles were defined based on the background population. There were 2374 participants in the first participation quintile, 4827 in the second, 5968 in the third, 7037 in the fourth, and 7890 in the fifth

For disease incidences, the numbers were quite similar across the background population and participants even without reweighting. In the case of CVD, the existing gap narrowed somewhat further by the reweighting; for cancer, it instead increased somewhat.

Since there is a definitional overlap between incidence and mortality, we also calculated incidences based only on nonfatal events (i.e., only hospitalizations). As it turned out, these incidences were virtually identical across participants and the background population even without reweighting (Table A1, supplement).

Table 2 (as well as Table A1, supplement) also shows numbers stratified on participation propensity quintiles. Across almost all the quintiles and outcomes, participants had more favorable outcomes than the background population. The finding reflects the limited success of the reweighting method: Shifting the distribution of the sample away from those with a high propensity to participate makes only a small difference since, throughout, there are unobserved factors that contribute to more favorable outcomes in participants.

**Fig. 2:**
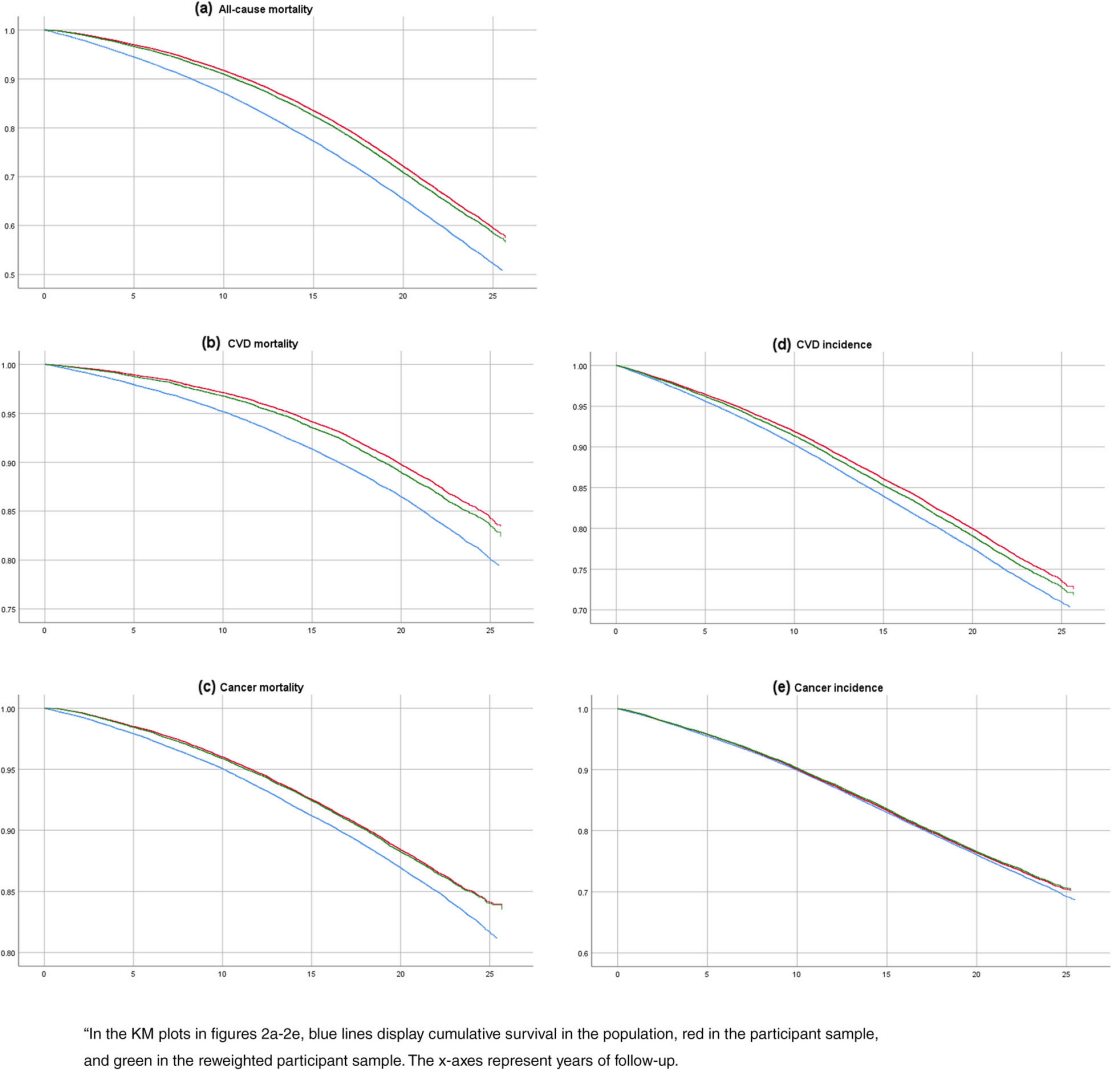
Kaplan-Meier (KM) plots

In Fig. 2, we show Kaplan Meier (KM) plots for the five outcomes considered, with separate lines representing cumulative incidences for the background population, the participant sample, and the reweighted participant sample. For all mortality outcomes, the graphs suggest that discrepancies in survival between participants and the background population appeared more or less immediately, and widened continuously over time. Throughout the time period, reweighting had only small effects. For disease incidences, numbers were quite similar for the background population, the participant sample, and the reweighted participant sample throughout the time period, especially so for cancer incidence.

In Table 3, we report associations (fully adjusted models) between all-cause mortality and the background variables, separately for the background population, the participant sample, and the reweighted participant sample. The corresponding results for cause-specific mortality and incidences are provided in Tables A2-A5 in the supplement. There were strong associations between the outcomes and background variables, especially age but also gender, socioeconomic status, and, in some cases, disease history.

**Table 3:** Associations between background characteristics and all-cause mortality – results from multivariable Cox regression models; the follow-up begins at baseline in 1991–1996 and ends at an event, death, emigration, or at the latest in the end of 2016

	Population (n = 71,447)	Participants (n = 28,096)	RHR	Participants, reweighted	RHR, weighted analysis
*Socio-demographics*					
Age					
40–45	1.00 (Ref.)	1.00 (Ref.)		1.00 (Ref.)	
46–50	1.27 (1.17–1.37)	1.21 (1.04–1.41)	0.95	1.18 (1.01–1.39)	0.93
51–55	1.95 (1.81–2.10)	2.02 (1.74–2.34)	1.04	1.96 (1.68–2.29)	1.01
56–60	3.16 (2.93–3.40)	3.41 (2.94–3.95)	1.08	3.37 (2.90–3.92)	1.07
61–64	4.33 (4.01–4.67)	5.03 (4.34–5.83)	1.16	5.07 (4.36–5.90)	1.17
65–67	6.51 (6.01–7.05)	7.72 (6.61–9.02)	1.19	7.84 (6.68–9.20)	1.20
Female	0.56 (0.55–0.58)	0.56 (0.54–0.59)	1.00	0.57 (0.54–0.59)	1.01
Country of birth					
Sweden	1.00 (Ref.)	1.00 (Ref.)		1.00 (Ref.)	
Other Nordic	1.03 (0.98–1.09)	1.01 (0.92–1.12)	0.98	1.02 (0.93–1.13)	0.99
Other EU15	0.80 (0.74–0.86)	0.82 (0.71–0.94)	1.02	0.83 (0.72–0.96)	1.05
Other EU	0.88 (0.83–0.94)	0.79 (0.68–0.91)	0.90	0.81 (0.69–0.93)	0.92
Other Europe	0.87 (0.83–0.93)	0.88 (0.73–1.06)	1.00	0.92 (0.76–1.11)	1.03
Outside Europe	0.79 (0.72–0.85)	0.68 (0.52–0.88)	0.86	0.68 (0.53–0.88)	0.87
Civil status					
Married	1.00 (Ref.)	1.00 (Ref.)		1.00 (Ref.)	
Unmarried	1.46 (1.41–1.51)	1.42 (1.32–1.53)	0.98	1.39 (1.29–1.50)	0.95
Divorced	1.38 (1.34–1.42)	1.32 (1.25–1.39)	0.96	1.32 (1.25–1.40)	0.96
Widowed	1.31 (1.26–1.37)	1.26 (1.18–1.36)	0.96	1.26 (1.17–1.35)	0.96
Education					
Primary	1.00 (Ref.)	1.00 (Ref.)		1.00 (Ref.)	
Short secondary	0.92 (0.89–0.94)	0.96 (0.92–1.01)	1.05	0.96 (0.92–1.01)	1.05
Long secondary	0.88 (0.85–0.92)	0.94 (0.88–1.00)	1.06	0.93 (0.87–1.00)	1.06
Tertiary	0.78 (0.75–0.81)	0.84 (0.79–0.89)	1.08	0.84 (0.79–0.90)	1.09
Employment status					
Employed	1.00 (Ref.)	1.00 (Ref.)		1.00 (Ref.)	
Unemployed	1.18 (1.12–1.25)	1.10 (0.99–1.22)	0.93	1.11 (1.00–1.23)	0.93
Sickness absence	1.41 (1.34–1.49)	1.41 (1.28–1.55)	1.00	1.43 (1.29–1.58)	1.01
Retired	1.61 (1.56–1.67)	1.60 (1.51–1.70)	0.99	1.58 (1.49–1.69)	0.98
Disposable income					
Quintile 1	1.00 (Ref.)	1.00 (Ref.)		1.00 (Ref.)	
Quintile 2	0.97 (0.93–1.00)	1.01 (0.94–1.08)	1.04	1.01 (0.94–1.08)	1.04
Quintile 3	0.88 (0.84–0.91)	0.94 (0.87–1.01)	1.07	0.94 (0.87–1.01)	1.07
Quintile 4	0.81 (0.78–0.85)	0.92 (0.86–0.99)	1.13	0.93 (0.86–1.00)	1.14
Quintile 5	0.71 (0.68–0.74)	0.81 (0.75–0.88)	1.14	0.82 (0.75–0.89)	1.16
*Disease history*					
Circulatory	1.50 (1.45–1.55)	1.46 (1.37–1.55)	0.97	1.48 (1.39–1.58)	0.99
Diabetes	2.41 (2.23–2.60)	2.68 (2.30–3.13)	1.11	2.66 (2.29–3.08)	1.10
Neoplasms	1.56 (1.49–1.63)	1.53 (1.42–1.66)	0.98	1.53 (1.40–1.67)	0.98
Respiratory	1.64 (1.55–1.73)	1.47 (1.32–1.64)	0.90	1.50 (1.33–1.70)	0.92
Digestive	1.16 (1.12–1.21)	1.15 (1.07–1.23)	0.99	1.14 (1.07–1.23)	0.98
Mental	1.92 (1.83–2.01)	2.19 (1.96–2.45)	1.14	2.23 (1.95–2.56)	1.16
R2	0.38	0.37		0.38	

The table reports hazard ratios (HRs) with 95% confidence intervals as well as ratios of hazard ratios (RHRs). R2 is the Royston & Sauerbrei R2 statistic for survival data [25]

Associations were generally similar across the background population, the participants, and the reweighted participants, with ratios of hazard ratios tending to be close to 1. However, the association between age and all-cause mortality as well as between age and CVD-related outcomes was larger in the participants than in the background population, and the association between mental illness and cancer-related outcomes was larger in the participants than in the background population. Throughout, it can be seen that the reweighting made virtually no difference for the associations.

## Discussion

Selective participation is generally a concern for studies based on voluntary participation. While, in principle, sampling methods such as quota sampling could be used to obtain cohorts that are similar to the full population with respect to observed background variables, these methods are rarely used in practice, implying that selective participation must be accounted for retrospectively. The MDC study is one example of a cohort with voluntary participation, and has been used in more than 100 published articles, typically without any reference to the lack of representativeness. The MDC study is also part of the European Prospective Investigation into Cancer and Nutrition Cohort (EPIC), one of the world’s largest cohort studies, with more than half a million participants. Assessing whether results based on these studies are representative of the underlying populations should therefore be of crucial interest. In this article, we set out to examine selection into the MDC study, the discrepancies in mortality, incidences, and associations across MDC participants and the background population, and to what extent reweighting the cohort with respect to observed background characteristics from registers allowed for the elimination of the discrepancies.

We found that the distributions of the background characteristics differed across MDC participants and the background population – not least in that socioeconomic status was higher among participants. Nevertheless, despite the high level of detailed background information, many determinants of selection into the cohort appeared to remain unobserved, as the ability of the background characteristics to discriminate between participants and non-participants was limited. Hence, for outcomes influenced by the same unobserved background characteristics that influenced selection, the reweighting method should have limited success.

While there were pronounced differences in mortality across participants and the background population, we found that reweighting based on observed background characteristics helped only little to narrow these gaps. Disease incidences and associations were more similar across participants and the background population already before reweighting. For CVD incidence, reweighting closed the existing gap somewhat, whereas for cancer incidence it actually increased. For associations, reweighting generally had little effect.

The particular finding that associations tended to be similar across participants and the background population, even without reweighting, is in line with several previous studies. For example, in the Norwegian Mother and Child Cohort Study and in the Danish National Birth Cohort, associations between risk factors and birth outcomes were generally similar across participating women and the full background populations of women giving birth, despite marked differences between participating women and the background populations in terms of background variables such as age and family status [4, 5]. Similarly, in a recent study based on another Swedish cohort (the Scania Public Health Cohort Study; SPHC), we documented that associations between different variables and mortality as well as drug purchases were relatively similar across participants, drop-outs, and the total cohort, although drop-out as such was strongly related to several variables including age, country of birth, smoking, socioeconomic status, and mortality [9]. In some contrast, however, another recent study compared participants in the UK Biobank with participants in the more representative Health Survey for England/Scottish Health Surveys (HSE-SHS) and found that the associations between CVD mortality and several risk factors (e.g., gender, glycated hemoglobin, and self-reported CVD) were substantially different across the two cohorts. Whether these discrepancies in associations could have been mitigated by reweighting based on observable characteristics was not examined.

Risk scores, such as the Framingham risk score for 10-year risk of CVD [26], are often estimated based on cohorts where self-selection may be an issue. If it is generally the case that associations between CVD and its risk factors in participant samples are similar to the corresponding associations in the background population, these risk scores may provide accurate conclusions about the relative risks across different population groups. As we have seen, however, the *levels* of outcomes may still vary across participants and background population, and a risk score calculated based on a self-selected cohort may therefore underestimate (or overestimate) risks for everyone.

## Conclusions

In conclusion, the results of the present study suggest that reweighting a health cohort with respect to sociodemographic and disease history variables that are commonly available in population registers is not necessarily sufficient to accurately estimate population-level outcomes, such as mortality. While this finding is somewhat discouraging, not least in light of the substantial and expanding literature on reweighing and generalizability [12–15, 22, 27], it should be emphasized that our results varied across the outcomes considered. For mortality and incidences, reweighting was at least somewhat helpful. Researchers using MDC data may thus be able to improve their validity to some extent by applying our weights, which we make available. On the other hand, several parameters were similar across participants and the background population already to begin with. We conclude that representativeness must be judged on a case-by-case basis [6]. Future research should examine the potential benefits of reweighting in other contexts, not least where associations differ markedly between unweighted participants and the background population.

## Supplementary Information

**Additional file 1: Table A1.** Incidences only including hospitalizations, not deaths (events per 10,000 person-years), in the background population and in the participant sample, before and after re-weighting; the follow-up begins at baseline in 1991–1996 and ends at an event, death, emigration, or at the latest in the end of 2016. **Table A2** Associations between background characteristics and CVD mortality – results from multivariable Cox regression models; the follow-up begins at baseline in 1991–1996 and ends at an event, death, emigration, or at the latest in the end of 2016. **Table A3** Associations between background characteristics and cancer mortality – results from multivariable Cox regression models; the follow-up begins at baseline in 1991–1996 and ends at an event, death, emigration, or at the latest in the end of 2016. **Table A4** Associations between background characteristics and CVD incidence – results from multivariable Cox regression models; the follow-up begins at baseline in 1991–1996 and ends at an event, death, emigration, or at the latest in the end of 2016. **Table A5** Associations between background characteristics and cancer incidence – results from multivariable Cox regression models; the follow-up begins at baseline in 1991–1996 and ends at an event, death, emigration, or at the latest in the end of 2016.

## Data Availability

The database used in this study is closed but researchers with an ethical approval from the Swedish Ethical Review Authority may contact the first author A.N. to gain access. We received access to the data via the register holders (the Malmö Diet and Cancer study at Lund University, Statistics Sweden, and the National Board of Health and Welfare in Sweden) after an ethical approval by the Regional Ethics Review Board in Lund.

## Abbreviations

CVD: Cardiovascular disease
ICD: International Classification of Diseases
IPPW: Inverse probability of participation weighting
KM: Kaplan Meier
MDC: Malmö Diet and Cancer
