# Supplementary material for: Can the validity of a cohort be improved by reweighting based on register data? Evidence from the Swedish MDC study
Source: BMC Public Health. 2020 Dec 17;20:1918. doi: 10.1186/s12889-020-10004-z (PMC7747383; doi:10.1186/s12889-020-10004-z)
Supplement: Supplementary file 1 — Additional file 1: Table A1. Incidences only including hospitalizations, not deaths (events per 10,000 person-years), in the background population and in the participant sample, before and after re-weighting; the follow-up begins at baseline in 1991–1996 and ends at an event, death, emigration, or at the latest in the end of 2016. Table A2 Associations between background characteristics and CVD mortality – results from multivariable Cox regression models; the follow-up begins at baseline in 1991–1996 and ends at an event, death, emigration, or at the latest in the end of 2016. Table A3 Associations between background characteristics and cancer mortality – results from multivariable Cox regression models; the follow-up begins at baseline in 1991–1996 and ends at an event, death, emigration, or at the latest in the end of 2016. Table A4 Associations between background characteristics and CVD incidence – results from multivariable Cox regression models; the follow-up begins at baseline in 1991–1996 and ends at an event, death, emigration, or at the latest in the end of 2016. Table A5 Associations between background characteristics and cancer incidence – results from multivariable Cox regression models; the follow-up begins at baseline in 1991–1996 and ends at an event, death, emigration, or at the latest in the end of 2016. [file 12889_2020_10004_MOESM1_ESM.docx]

**Table A1** Incidences only including hospitalizations, not deaths (events per 10,000 person-years), in the background population and in the participant sample, before and after re-weighting; the follow-up begins at baseline in 1991-1996 and ends at an event, death, emigration, or at the latest in the end of 2016

|  | Population (n=71,447) | Participants (n=28,096) | Participants, re-weighted |
| --- | --- | --- | --- |
| CVD hospitalization |  |  |  |
| Everyone | 102 | 97 | 99 |
| Participation quintile 1 | 106 | 101 | 101 |
| Participation quintile 2 | 118 | 117 | 116 |
| Participation quintile 3 | 108 | 104 | 104 |
| Participation quintile 4 | 92 | 87 | 87 |
| Participation quintile 5 | 90 | 88 | 88 |
| Cancer hospitalization |  |  |  |
| Everyone | 128 | 128 | 126 |
| Participation quintile 1 | 111 | 115 | 112 |
| Participation quintile 2 | 139 | 133 | 132 |
| Participation quintile 3 | 130 | 127 | 127 |
| Participation quintile 4 | 127 | 127 | 127 |
| Participation quintile 5 | 134 | 129 | 129 |

Participation quintiles were defined based on the background population. There were 2,374 participants in the first participation quintile, 4,827 in the second, 5,968 in the third, 7,037 in the fourth, and 7,890 in the fifth.

**Table A2** Associations between background characteristics and CVD mortality – results from multivariable Cox regression models; the follow-up begins at baseline in 1991-1996 and ends at an event, death, emigration, or at the latest in the end of 2016

|  | Population (n=71,447) | Participants (n=28,096) | RHR | Participants, re-weighted | RHR, weighted analysis |
| --- | --- | --- | --- | --- | --- |
| *Socio-demographics* |  |  |  |  |  |
| Age |  |  |  |  |  |
| 40-45 | 1.00 (Ref.) | 1.00 (Ref.) |  | 1.00 (Ref.) |  |
| 46-50 | 1.74 (1.45-2.07) | 1.67 (1.13-2.48) | 0.96 | 1.57 (1.05-2.34) | 0.90 |
| 51-55 | 2.90 (2.44-3.45) | 3.27 (2.23-4.81) | 1.13 | 3.17 (2.14-4.69) | 1.09 |
| 56-60 | 5.29 (4.46-6.28) | 6.35 (4.35-9.26) | 1.20 | 6.45 (4.39-9.47) | 1.22 |
| 61-64 | 7.92 (6.67-9.41) | 10.30 (7.06-15.02) | 1.30 | 10.64 (7.22-15.67) | 1.34 |
| 65-67 | 12.59 (10.55-15.03) | 16.68 (11.34-24.54) | 1.32 | 17.25 (11.60-25.66) | 1.37 |
| Female | 0.48 (0.46-0.50) | 0.44 (0.41-0.48) | 0.92 | 0.44 (0.40-0.48) | 0.92 |
| Country of birth |  |  |  |  |  |
| Sweden | 1.00 (Ref.) | 1.00 (Ref.) |  | 1.00 (Ref.) |  |
| Other Nordic | 0.94 (0.86-1.03) | 0.93 (0.78-1.11) | 0.99 | 0.95 (0.79-1.14) | 1.00 |
| Other EU15 | 0.83 (0.73-0.94) | 0.81 (0.64-1.04) | 0.99 | 0.83 (0.65-1.07) | 1.01 |
| Other EU | 0.94 (0.86-1.04) | 0.94 (0.75-1.18) | 0.99 | 0.96 (0.76-1.22) | 1.02 |
| Other Europe | 0.89 (0.80-0.98) | 0.96 (0.70-1.33) | 1.09 | 1.00 (0.73-1.39) | 1.13 |
| Outside Europe | 0.79 (0.68-0.91) | 0.62 (0.39-1.01) | 0.79 | 0.60 (0.36-1.00) | 0.77 |
| Civil status |  |  |  |  |  |
| Married | 1.00 (Ref.) | 1.00 (Ref.) |  | 1.00 (Ref.) |  |
| Unmarried | 1.63 (1.54-1.74) | 1.65 (1.46-1.86) | 1.01 | 1.61 (1.41-1.83) | 0.98 |
| Divorced | 1.47 (1.40-1.55) | 1.41 (1.28-1.55) | 0.96 | 1.41 (1.28-1.56) | 0.96 |
| Widowed | 1.39 (1.30-1.49) | 1.41 (1.25-1.59) | 1.01 | 1.40 (1.23-1.59) | 1.01 |
| Education |  |  |  |  |  |
| Primary | 1.00 (Ref.) | 1.00 (Ref.) |  | 1.00 (Ref.) |  |
| Short secondary | 0.90 (0.86-0.94) | 0.94 (0.86-1.02) | 1.04 | 0.94 (0.86-1.02) | 1.05 |
| Long secondary | 0.88 (0.82-0.94) | 0.98 (0.88-1.10) | 1.12 | 0.97 (0.86-1.09) | 1.11 |
| Tertiary | 0.75 (0.70-0.80) | 0.87 (0.78-0.97) | 1.16 | 0.89 (0.79-0.99) | 1.18 |
| Employment status |  |  |  |  |  |
| Employed | 1.00 (Ref.) | 1.00 (Ref.) |  | 1.00 (Ref.) |  |
| Unemployed | 1.18 (1.08-1.29) | 1.11 (0.92-1.33) | 0.94 | 1.12 (0.93-1.35) | 0.95 |
| Sickness absence | 1.47 (1.34-1.61) | 1.54 (1.30-1.83) | 1.05 | 1.54 (1.28-1.85) | 1.05 |
| Retired | 1.71 (1.60-1.81) | 1.78 (1.60-1.98) | 1.04 | 1.78 (1.59-1.98) | 1.04 |
| Disposable income |  |  |  |  |  |
| Quintile 1 | 1.00 (Ref.) | 1.00 (Ref.) |  | 1.00 (Ref.) |  |
| Quintile 2 | 0.95 (0.90-1.01) | 0.93 (0.83-1.04) | 0.98 | 0.93 (0.83-1.05) | 0.98 |
| Quintile 3 | 0.86 (0.81-0.92) | 0.83 (0.74-0.94) | 0.97 | 0.83 (0.73-0.94) | 0.96 |
| Quintile 4 | 0.78 (0.72-0.84) | 0.83 (0.73-0.95) | 1.07 | 0.82 (0.72-0.94) | 1.06 |
| Quintile 5 | 0.63 (0.58-0.68) | 0.65 (0.56-0.75) | 1.04 | 0.65 (0.56-0.76) | 1.04 |
| *Disease history* |  |  |  |  |  |
| Circulatory | 2.18 (2.07-2.29) | 1.97 (1.79-2.16) | 0.91 | 2.01 (1.82-2.22) | 0.92 |
| Diabetes | 2.88 (2.56-3.25) | 3.33 (2.62-4.22) | 1.15 | 3.37 (2.63-4.32) | 1.17 |
| Neoplasms | 1.17 (1.07-1.28) | 1.21 (1.04-1.42) | 1.04 | 1.23 (1.05-1.44) | 1.05 |
| Respiratory | 1.41 (1.28-1.55) | 1.35 (1.12-1.64) | 0.96 | 1.36 (1.10-1.68) | 0.96 |
| Digestive | 1.11 (1.04-1.18) | 1.09 (0.97-1.22) | 0.98 | 1.07 (0.94-1.21) | 0.96 |
| Mental | 1.82 (1.68-1.98) | 2.27 (1.88-2.73) | 1.24 | 2.26 (1.82-2.81) | 1.24 |
| R2 | 0.48 | 0.48 |  | 0.49 |  |

The table reports hazard ratios (HRs) with 95% confidence intervals as well as ratios of hazard ratios (RHRs). R2 is the Royston & Sauerbrei R2 statistic for survival data.^1^

**Table A3** Associations between background characteristics and cancer mortality – results from multivariable Cox regression models; the follow-up begins at baseline in 1991-1996 and ends at an event, death, emigration, or at the latest in the end of 2016

|  | Population  (n=71,447) | Participants (n=28,096) | RHR | Participants, re-weighted | RHR, weighted analysis |
| --- | --- | --- | --- | --- | --- |
| *Socio-demographics* |  |  |  |  |  |
| Age |  |  |  |  |  |
| 40-45 | 1.00 (Ref.) | 1.00 (Ref.) |  | 1.00 (Ref.) |  |
| 46-50 | 1.11 (0.99-1.24) | 1.13 (0.92-1.38) | 1.02 | 1.13 (0.92-1.39) | 1.02 |
| 51-55 | 1.62 (1.45-1.80) | 1.73 (1.42-2.10) | 1.07 | 1.69 (1.38-2.07) | 1.05 |
| 56-60 | 2.33 (2.09-2.59) | 2.44 (2.01-2.96) | 1.05 | 2.40 (1.97-2.92) | 1.03 |
| 61-64 | 2.84 (2.54-3.17) | 3.07 (2.52-3.73) | 1.08 | 3.04 (2.49-3.72) | 1.07 |
| 65-67 | 3.54 (3.13-4.00) | 3.76 (3.02-4.68) | 1.06 | 3.78 (3.02-4.74) | 1.07 |
| Female | 0.60 (0.58-0.63) | 0.63 (0.58-0.68) | 1.04 | 0.63 (0.58-0.69) | 1.05 |
| Country of birth |  |  |  |  |  |
| Sweden | 1.00 (Ref.) | 1.00 (Ref.) |  | 1.00 (Ref.) |  |
| Other Nordic | 1.07 (0.98-1.18) | 0.92 (0.78-1.10) | 0.86 | 0.93 (0.78-1.11) | 0.87 |
| Other EU15 | 0.79 (0.69-0.90) | 0.82 (0.65-1.03) | 1.04 | 0.84 (0.66-1.07) | 1.07 |
| Other EU | 0.80 (0.72-0.89) | 0.70 (0.54-0.89) | 0.87 | 0.70 (0.54-0.91) | 0.88 |
| Other Europe | 0.84 (0.75-0.93) | 0.87 (0.64-1.18) | 1.04 | 0.92 (0.67-1.25) | 1.10 |
| Outside Europe | 0.72 (0.62-0.84) | 0.69 (0.45-1.03) | 0.95 | 0.71 (0.47-1.08) | 0.98 |
| Civil status |  |  |  |  |  |
| Married | 1.00 (Ref.) | 1.00 (Ref.) |  | 1.00 (Ref.) |  |
| Unmarried | 1.17 (1.10-1.25) | 1.23 (1.09-1.39) | 1.05 | 1.19 (1.05-1.35) | 1.02 |
| Divorced | 1.18 (1.12-1.25) | 1.17 (1.07-1.28) | 0.99 | 1.18 (1.07-1.29) | 0.99 |
| Widowed | 1.18 (1.09-1.27) | 1.19 (1.05-1.35) | 1.01 | 1.17 (1.03-1.34) | 1.00 |
| Education |  |  |  |  |  |
| Primary | 1.00 (Ref.) | 1.00 (Ref.) |  | 1.00 (Ref.) |  |
| Short secondary | 0.94 (0.89-0.98) | 0.98 (0.90-1.06) | 1.05 | 0.97 (0.89-1.06) | 1.04 |
| Long secondary | 0.89 (0.83-0.96) | 0.91 (0.81-1.02) | 1.02 | 0.90 (0.80-1.01) | 1.01 |
| Tertiary | 0.78 (0.73-0.84) | 0.83 (0.74-0.92) | 1.05 | 0.83 (0.75-0.92) | 1.06 |
| Employment status |  |  |  |  |  |
| Employed | 1.00 (Ref.) | 1.00 (Ref.) |  | 1.00 (Ref.) |  |
| Unemployed | 1.11 (1.01-1.21) | 1.02 (0.86-1.21) | 0.92 | 1.03 (0.87-1.22) | 0.93 |
| Sickness absence | 1.26 (1.15-1.38) | 1.28 (1.10-1.50) | 1.02 | 1.32 (1.12-1.55) | 1.05 |
| Retired | 1.27 (1.20-1.36) | 1.33 (1.20-1.47) | 1.04 | 1.32 (1.19-1.47) | 1.04 |
| Disposable income |  |  |  |  |  |
| Quintile 1 | 1.00 (Ref.) | 1.00 (Ref.) |  | 1.00 (Ref.) |  |
| Quintile 2 | 1.04 (0.97-1.11) | 1.12 (0.99-1.26) | 1.08 | 1.13 (1.00-1.28) | 1.09 |
| Quintile 3 | 0.98 (0.92-1.06) | 1.07 (0.94-1.20) | 1.08 | 1.09 (0.96-1.23) | 1.11 |
| Quintile 4 | 0.94 (0.87-1.01) | 1.02 (0.90-1.16) | 1.09 | 1.04 (0.91-1.19) | 1.11 |
| Quintile 5 | 0.88 (0.82-0.96) | 1.03 (0.89-1.18) | 1.16 | 1.05 (0.92-1.21) | 1.19 |
| *Disease history* |  |  |  |  |  |
| Circulatory | 1.07 (0.99-1.15) | 1.11 (0.99-1.25) | 1.05 | 1.14 (1.01-1.29) | 1.06 |
| Diabetes | 0.94 (0.75-1.17) | 1.11 (0.75-1.63) | 1.18 | 1.07 (0.71-1.59) | 1.14 |
| Neoplasms | 2.42 (2.26-2.60) | 2.16 (1.92-2.42) | 0.89 | 2.14 (1.89-2.42) | 0.88 |
| Respiratory | 1.30 (1.16-1.45) | 1.20 (0.98-1.47) | 0.93 | 1.23 (0.99-1.52) | 0.95 |
| Digestive | 1.08 (1.00-1.16) | 1.11 (0.98-1.25) | 1.03 | 1.11 (0.98-1.26) | 1.03 |
| Mental | 1.44 (1.30-1.59) | 1.86 (1.53-2.27) | 1.29 | 1.92 (1.56-2.36) | 1.33 |
| R2 | 0.22 | 0.22 |  | 0.22 |  |

The table reports hazard ratios (HRs) with 95% confidence intervals as well as ratios of hazard ratios (RHRs). R2 is the Royston & Sauerbrei R2 statistic for survival data.^1^

**Table A4** Associations between background characteristics and CVD incidence – results from multivariable Cox regression models; the follow-up begins at baseline in 1991-1996 and ends at an event, death, emigration, or at the latest in the end of 2016

|  | Population  (n=69,583) | Participants (n=27,491) | RHR | Participants, re-weighted | RHR, weighted analysis |
| --- | --- | --- | --- | --- | --- |
| *Socio-demographics* |  |  |  |  |  |
| Age |  |  |  |  |  |
| 40-45 | 1.00 (Ref.) | 1.00 (Ref.) |  | 1.00 (Ref.) |  |
| 46-50 | 1.51 (1.37-1.67) | 1.67 (1.38-2.02) | 1.11 | 1.63 (1.34-1.98) | 1.08 |
| 51-55 | 2.16 (1.96-2.38) | 2.48 (2.06-2.99) | 1.15 | 2.41 (1.99-2.92) | 1.12 |
| 56-60 | 3.03 (2.75-3.34) | 3.67 (3.05-2.99) | 1.21 | 3.64 (3.01-4.40) | 1.20 |
| 61-64 | 4.01 (3.63-4.43) | 4.77 (3.95-5.75) | 1.19 | 4.78 (3.95-5.80) | 1.19 |
| 65-67 | 5.24 (4.71-5.83) | 6.49 (5.31-7.94) | 1.24 | 6.46 (5.25-7.94) | 1.23 |
| Female | 0.51 (0.49-0.52) | 0.47 (0.44-0.50) | 0.94 | 0.47 (0.44-0.50) | 0.92 |
| Country of birth |  |  |  |  |  |
| Sweden | 1.00 (Ref.) | 1.00 (Ref.) |  | 1.00 (Ref.) |  |
| Other Nordic | 0.98 (0.91-1.05) | 1.02 (0.89-1.16) | 1.04 | 1.03 (0.90-1.18) | 1.05 |
| Other EU15 | 0.86 (0.78-1.05) | 0.96 (0.81-1.13) | 1.12 | 0.97 (0.82-1.14) | 1.13 |
| Other EU | 0.98 (0.91-1.06) | 1.05 (0.81-1.13) | 1.07 | 1.04 (0.88-1.23) | 1.06 |
| Other Europe | 0.94 (0.87-1.02) | 0.86 (0.69-1.09) | 0.92 | 0.88 (0.78-1.35) | 0.93 |
| Outside Europe | 0.95 (0.86-1.06) | 1.04 (0.79-1.35) | 1.09 | 1.03 (0.78-1.35) | 1.08 |
| Civil status |  |  |  |  |  |
| Married | 1.00 (Ref.) | 1.00 (Ref.) |  | 1.00 (Ref.) |  |
| Unmarried | 1.16 (1.10-1.22) | 1.17 (1.06-1.28) | 1.01 | 1.16 (1.05-1.33) | 1.00 |
| Divorced | 1.27 (1.22-1.32) | 1.23 (1.14-1.32) | 0.97 | 1.24 (1.15-1.33) | 0.98 |
| Widowed | 1.21 (1.14-1.29) | 1.15 (1.04-1.28) | 0.95 | 1.17 (1.06-1.30) | 0.96 |
| Education |  |  |  |  |  |
| Primary | 1.00 (Ref.) | 1.00 (Ref.) |  | 1.00 (Ref.) |  |
| Short secondary | 0.92 (0.89-0.96) | 0.94 (0.88-1.00) | 1.02 | 0.94 (0.88-1.00) | 1.02 |
| Long secondary | 0.91 (0.86-0.96) | 0.96 (0.89-1.05) | 1.06 | 0.97 (0.89-1.06) | 1.07 |
| Tertiary | 0.72 (0.68-0.76) | 0.79 (0.73-0.86) | 1.09 | 0.81 (0.74-0.88) | 1.12 |
| Employment status |  |  |  |  |  |
| Employed | 1.00 (Ref.) | 1.00 (Ref.) |  | 1.00 (Ref.) |  |
| Unemployed | 1.12 (1.04-1.20) | 1.04 (0.92-1.18) | 0.94 | 1.04 (0.92-1.19) | 0.93 |
| Sickness absence | 1.29 (1.20-1.39) | 1.29 (1.14-1.46) | 1.00 | 1.27 (1.12-1.45) | 0.99 |
| Retired | 1.38 (1.31-1.44) | 1.34 (1.24-1.45) | 0.97 | 1.33 (1.22-1.45) | 0.97 |
| Disposable income |  |  |  |  |  |
| Quintile 1 | 1.00 (Ref.) | 1.00 (Ref.) |  | 1.00 (Ref.) |  |
| Quintile 2 | 0.98 (0.94-1.03) | 0.94 (0.86-1.03) | 0.96 | 0.95 (0.86-1.04) | 0.96 |
| Quintile 3 | 0.95 (0.90-1.00) | 0.86 (0.79-0.95) | 0.91 | 0.84 (0.76-0.92) | 0.89 |
| Quintile 4 | 0.88 (0.83-0.94) | 0.88 (0.80-0.97) | 1.00 | 0.86 (0.78-0.95) | 0.97 |
| Quintile 5 | 0.81 (0.76-0.87) | 0.75 (0.67-0.83) | 0.92 | 0.74 (0.66-0.82) | 0.90 |
| *Disease history* |  |  |  |  |  |
| Circulatory | 1.71 (1.62-1.80) | 1.61 (1.47-1.77) | 0.94 | 1.61 (1.47-1.77) | 0.94 |
| Diabetes | 2.93 (2.63-3.26) | 3.12 (2.56-3.80) | 1.06 | 3.14 (2.56-3.86) | 1.07 |
| Neoplasms | 1.09 (1.01-1.17) | 1.05 (0.93-1.19) | 0.96 | 1.08 (0.95-1.22) | 0.99 |
| Respiratory | 1.27 (1.17-1.39) | 1.27 (1.09-1.49) | 1.00 | 1.28 (1.08-1.51) | 1.00 |
| Digestive | 1.07 (1.01-1.14) | 1.03 (0.94-1.14) | 0.96 | 1.03 (0.93-1.14) | 0.96 |
| Mental | 1.39 (1.28-1.50) | 1.53 (1.30-1.80) | 1.10 | 1.50 (1.26-1.79) | 1.08 |
| R2 | 0.29 | 0.29 |  | 0.29 |  |

The table reports hazard ratios (HRs) with 95% confidence intervals as well as ratios of hazard ratios (RHRs). R2 is the Royston & Sauerbrei R2 statistic for survival data.^1^

**Table A5** Associations between background characteristics and cancer incidence – results from multivariable Cox regression models; the follow-up begins at baseline in 1991-1996 and ends at an event, death, emigration, or at the latest in the end of 2016

|  | Population  (n=69,362) | Participants (n=27,288) | RHR | Participants, re-weighted | RHR, weighted analysis |
| --- | --- | --- | --- | --- | --- |
| *Socio-demographics* |  |  |  |  |  |
| Age |  |  |  |  |  |
| 40-45 | 1.00 (Ref.) | 1.00 (Ref.) |  | 1.00 (Ref.) |  |
| 46-50 | 1.04 (0.96-1.11) | 1.10 (0.98-1.24) | 1.06 | 1.12 (1.00-1.27) | 1.09 |
| 51-55 | 1.39 (1.30-1.50) | 1.43 (1.27-1.61) | 1.03 | 1.45 (1.28-1.63) | 1.04 |
| 56-60 | 1.69 (1.57-1.81) | 1.72 (1.53-1.93) | 1.02 | 1.72 (1.53-1.94) | 1.02 |
| 61-64 | 1.96 (1.82-2.11) | 2.00 (1.78-2.26) | 1.03 | 2.01 (1.77-2.27) | 1.03 |
| 65-67 | 2.29 (2.10-2.50) | 2.33 (2.01-2.69) | 1.02 | 2.33 (2.01-2.70) | 1.02 |
| Female | 0.73 (0.71-0.76) | 0.75 (0.71-0.79) | 1.02 | 0.75 (0.71-0.80) | 1.03 |
| Country of birth |  |  |  |  |  |
| Sweden | 1.00 (Ref.) | 1.00 (Ref.) |  | 1.00 (Ref.) |  |
| Other Nordic | 1.04 (0.96-1.11) | 0.97 (0.85-1.10) | 0.94 | 1.00 (0.88-1.14) | 0.96 |
| Other EU15 | 0.87 (0.79-0.96) | 0.95 (0.81-1.11) | 1.09 | 0.95 (0.81-1.11) | 1.09 |
| Other EU | 0.82 (0.76-0.89) | 0.82 (0.69-0.96) | 1.00 | 0.84 (0.71-0.99) | 1.02 |
| Other Europe | 0.84 (0.77-0.91) | 0.81 (0.66-1.01) | 0.98 | 0.82 (0.66-1.02) | 0.99 |
| Outside Europe | 0.68 (0.60-0.76) | 0.72 (0.54-0.95) | 1.06 | 0.69 (0.52-0.93) | 1.03 |
| Civil status |  |  |  |  |  |
| Married | 1.00 (Ref.) | 1.00 (Ref.) |  | 1.00 (Ref.) |  |
| Unmarried | 1.04 (0.99-1.10) | 1.08 (0.99-1.18) | 1.04 | 1.07 (0.98-1.17) | 1.03 |
| Divorced | 1.06 (1.02-1.11) | 1.05 (0.98-1.13) | 0.99 | 1.06 (0.99-1.13) | 0.99 |
| Widowed | 1.06 (1.00-1.13) | 1.07 (0.97-1.18) | 1.01 | 1.06 (0.96-1.17) | 1.00 |
| Education |  |  |  |  |  |
| Primary | 1.00 (Ref.) | 1.00 (Ref.) |  | 1.00 (Ref.) |  |
| Short secondary | 0.96 (0.93-1.00) | 0.98 (0.92-1.04) | 1.02 | 0.98 (0.92-1.04) | 1.02 |
| Long secondary | 0.92 (0.88-0.97) | 0.96 (0.88-1.04) | 1.04 | 0.95 (0.87-1.03) | 1.03 |
| Tertiary | 0.89 (0.85-0.94) | 0.94 (0.88-1.01) | 1.05 | 0.94 (0.87-1.01) | 1.05 |
| Employment status |  |  |  |  |  |
| Employed | 1.00 (Ref.) | 1.00 (Ref.) |  | 1.00 (Ref.) |  |
| Unemployed | 0.97 (0.91-1.04) | 0.90 (0.80-1.02) | 0.93 | 0.90 (0.80-1.02) | 0.93 |
| Sickness absence | 1.06 (0.98-1.14) | 1.16 (1.03-1.30) | 1.09 | 1.16 (1.03-1.31) | 1.10 |
| Retired | 1.09 (1.04-1.14) | 1.07 (0.99-1.16) | 0.99 | 1.08 (1.00-1.17) | 0.99 |
| Disposable income |  |  |  |  |  |
| Quintile 1 | 1.00 (Ref.) | 1.00 (Ref.) |  | 1.00 (Ref.) |  |
| Quintile 2 | 1.06 (1.00-1.11) | 1.08 (0.98-1.18) | 1.02 | 1.08 (0.99-1.18) | 1.02 |
| Quintile 3 | 1.04 (0.98-1.10) | 1.06 (0.97-1.16) | 1.02 | 1.07 (0.98-1.17) | 1.03 |
| Quintile 4 | 1.02 (0.97-1.08) | 1.03 (0.94-1.13) | 1.00 | 1.05 (0.95-1.15) | 1.02 |
| Quintile 5 | 1.01 (0.95-1.07) | 1.05 (0.95-1.16) | 1.04 | 1.08 (0.97-1.19) | 1.07 |
| *Disease history* |  |  |  |  |  |
| Circulatory | 1.12 (1.06-1.18) | 1.04 (0.95-1.14) | 0.97 | 1.07 (0.98-1.18) | 0.96 |
| Diabetes | 0.97 (0.82-1.15) | 1.01 (0.75-1.35) | 1.04 | 1.05 (0.78-1.42) | 1.09 |
| Neoplasms | 1.09 (0.99-1.21) | 1.11 (0.95-1.30) | 1.01 | 1.11 (0.94-1.30) | 1.01 |
| Respiratory | 1.20 (1.10-1.32) | 1.22 (1.05-1.41) | 1.01 | 1.23 (1.06-1.44) | 1.03 |
| Digestive | 1.06 (1.00-1.13) | 1.12 (1.02-1.23) | 1.05 | 1.12 (1.02-1.23) | 1.05 |
| Mental | 1.19 (1.10-1.30) | 1.41 (1.20-1.66) | 1.18 | 1.45 (1.23-1.71) | 1.21 |
| R2 | 0.09 | 0.08 |  | 0.08 |  |

The table reports hazard ratios (HRs) with 95% confidence intervals as well as ratios of hazard ratios (RHRs). R2 is the Royston & Sauerbrei R2 statistic for survival data.^1^

**Reference**

1. Royston, P. & Sauerbrei, W. A new measure of prognistic separation in survival data. *Stat Med* **23**, 723–48 (2004).
